# Cerebral venous biomarkers and veno-arterial gradients: untapped resources in Alzheimer’s disease

**DOI:** 10.3389/fneur.2023.1295122

**Published:** 2024-01-04

**Authors:** Paul V. Suhocki, P. Murali Doraiswamy

**Affiliations:** ^1^Duke University Hospital, Durham, NC, United States; ^2^School of Medicine, Duke University, Durham, NC, United States; ^3^Division of Interventional Radiology, Department of Radiology, Duke University Hospital, Durham, NC, United States; ^4^Duke Institute for Brain Sciences, School of Medicine, Duke University, Durham, NC, United States; ^5^Department of Psychiatry and Behavioral Sciences, School of Medicine, Duke University, Durham, NC, United States

**Keywords:** Alzheimer’s disease, biomarkers, radiology, venous, arterial

## Abstract

Blood based biomarkers (BBB) derived from forearm veins for estimating brain changes is becoming ubiquitous in Alzheimer’s Disease (AD) research and could soon become standard in routine clinical diagnosis. However, there are many peripheral sources of contamination through which concentrations of these metabolites can be raised or lowered after leaving the brain and entering the central venous pool. This raises the issue of potential false conclusions that could lead to erroneous diagnosis or research findings. We propose the use of simultaneous sampling of internal jugular venous and arterial blood to calculate veno-arterial gradient, which can reveal either a surplus or a deficit of metabolites exiting the brain. Methods for sampling internal jugular venous and arterial blood are described along with examples of the use of the veno-arterial gradient in non-AD brain research. Such methods in turn could help better establish the accuracy of forearm venous biomarkers.

## Perspective article

The “streetlight effect” is a metaphor commonly used to describe a human convenience bias. To quote Noam Chomsky, “Science is a bit like the joke about the [man] who is looking under a lamppost for a key that he has lost on the other side of the street, because that’s where the light is. It has no other choice.” The search for Alzheimer’s Disease (AD) biomarkers in blood sourced from peripheral veins, while convenient, may be a victim of this effect. We have ignored what may lie in the darkness.

The forearm vein is an easily accessible source of blood and the practice of using this site for blood collection has become ubiquitous in AD blood-based biomarker and ‘omics diagnostic and therapeutic research. There are many reasons to celebrate the advent of ultrasensitive assays and newer blood biomarkers; they offer convenience, safety, repeat sampling, ability to scale and point of care and home-based applications. However, there is an inherent limitation in using forearm venous blood for brain biomarkers. In a recent review of blood-based biomarkers Hampel et al. noted that individuals with documented brain amyloid pathology had reductions of approximately 10–15% in plasma Aß42/Aß40 compared with reductions of 50% in CSF Aß42/Aß40 (1). The authors speculated that the low effect size of the plasma Aß ratio could be a result of peripherally produced Aß diluting the brain-derived Aß and reducing the dynamic range of the measure. This would seem likely as platelets can produce more than 90% of blood Aβ (2). Biomarker dilution in peripheral venous blood samples is not unique to AD research. Endocrine researchers observed a twelvefold reduction in adrenocorticotropin levels in blood collected from a forearm vein compared with blood drawn from the inferior petrosal sinuses of the brain (3). Other factors confounding the interpretation of peripheral plasma biomarker concentrations include body mass index and renal function which can alter NfL, pTau and GFAP blood levels (4, 5).

Concentrations of biomarkers can also change as they are either consumed or produced in metabolically active tissues in the periphery. Blood concentrations of glutamine, alanine, malate, phenylalanine, lactate, sialic acid and succinate drawn from forearm veins draining musculoskeletal tissues of the forearm are significantly lower than in arteries carrying blood to those tissues; concentrations of serine, aspartate and glutamate in forearm veins are significantly higher than those in forearm arteries (6).

**Figure 1:**
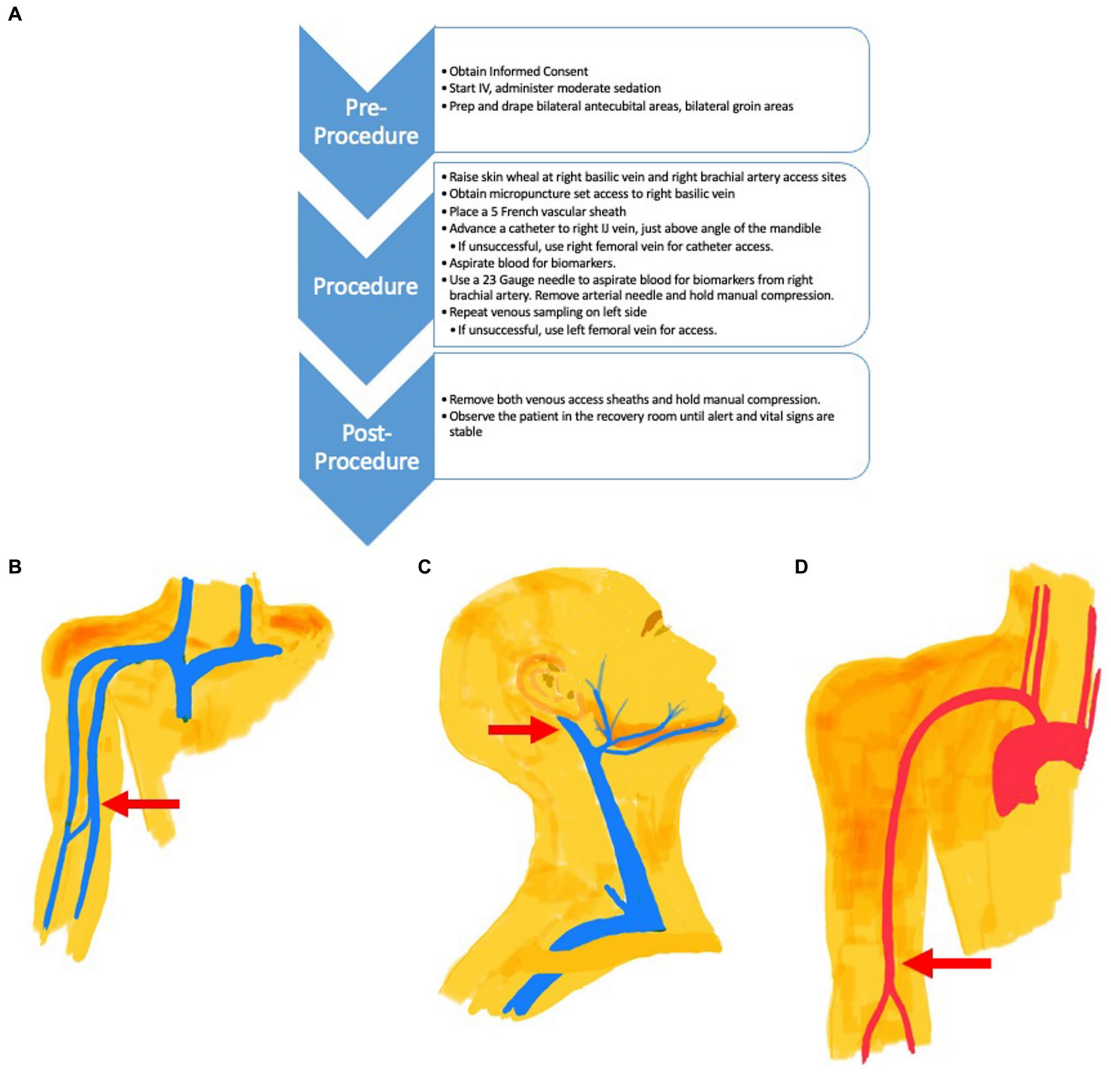
**(A)** Procedure Flow Chart for Venous and Arterial Access. **(B)** The venous catheter access site is located in the Basilic Vein (Red Arrow) just above the elbow. The catheter is directed to the internal jugular vein **(C)** for blood collection. **(C)** The tip of the venous catheter placed via the arm basilic vein is directed to the internal jugular vein (Red Arrow) for venous blood collection, just above the facial vein branch. **(D)** Arterial needle access site (Red Arrow) is located in the brachial artery, just above the elbow. Blood is collected from the needle directly at this site. A catheter is not used.

Based on these observations, we believe that the value of blood-based biomarkers of brain function may be enhanced, during initial validation, by changing the site from where the blood is drawn and by adding an arterial blood draw (Figure 1A). One sample of blood is collected from the internal jugular vein, not directly but through the tip of a catheter guided to that location from a basilic vein access point just above the elbow (Figure 1B). Collecting venous blood above the angle of the jaw ensures that the sample is not diluted by inflow from the facial vein below this level (Figure 1C). Bilateral venous sampling is recommended as venous drainage from the hippocampi can be asymmetric. A blood sample is also collected directly from the brachial artery using a needle (Figure 1D). The difference between the venous concentration and the arterial concentration is the “veno-arterial gradient” or “veno-arterial difference” which should represent the brain’s biomarker contribution to venous blood. In the case of Aß42, this should result in a lower Aß42 value, improving the effect size of the plasma Aß42/Aß40, with the ratio more closely approximating that found in CSF. This technique should also be able to provide more representative concentrations for other brain biomarkers.

The veno-arterial gradient can also be used to further our understanding of AD brain metabolism as it provides the difference in concentrations of metabolites entering and exiting the brain. Use of the gradient has already revealed the brain’s role in obesity (7), lipopolysaccharide-induced systemic inflammation (8), traumatic brain injury (9) and treatment-refractory Depression (10). Non-neurological scientists have used venous–arterial gradients to elucidate umbilical arterial and venous cord blood differences (11), cancer metabolic consumption (12), mammary gland milk synthesis metabolic pathways (13) and pharmacokinetic modeling (14).

In the study of AD, Hoyer et al. utilized the brain veno-arterial ammonia gradient to demonstrate a surplus of ammonia in blood exiting the brain in AD subjects compared with a deficit of ammonia in blood exiting the brain in subjects with normal cognition, concluding that the AD brain has an endogenous source of ammonia (15). Researchers since discovered a reduction of glutamine synthetase and an elevation of adenosine monophosphate in the AD brain, both contributing to high brain levels of neurotoxic ammonia. Perhaps further AD research using brain veno-arterial gradients will reveal a metabolite that can be easily assayed in the clinic for prompt diagnosis of AD, rapid enrollment into clinical trials or for following patient progress on an AD therapeutic.

Collecting blood in the aforementioned manner may seem overly complicated but is actually rather straightforward to perform for an Interventional Radiologist who can be found in all tertiary care medical centers and most community hospitals. Interventional Radiologists are skilled in the collection of blood from the adrenal veins and inferior petrosal sinus of the brain for patients with Cushing’s Disease and Conn’s Syndrome (16), the renal veins for patients with hypertension secondary to hyper-reninism (17), the hepatic veins during segmental calcium gluconate stimulation of the pancreas in patients with hyper-insulinism (18) and the inferior thyroid veins in patients with ectopic sources of parathyroid hormone (19).

We are not advocating the immediate implementation of these blood sampling techniques into routine AD care but are instead recommending that the AD community explore their use in validating the accuracy of blood-based biomarkers that more closely reflect contributions from the brain and for furthering the understanding of AD brain metabolism.

Future work may simplify these blood sampling procedures by demonstrating equivalence between unilateral and bilateral internal jugular venous blood sampling and demonstrating that low internal jugular vein blood sampling, below the inflow from the facial vein, provides adequate samples for brain biomarker assay. If low internal jugular vein sampling can provide sufficient samples, a simple needle directed blood draw directly from the internal jugular vein can be performed under ultrasound guidance without the need for fluoroscopic guidance of a catheter placed in the arm.

There are some logistic limitations to adopting the described blood sampling techniques. Cerebral venous blood draws may require an interventional radiologist. Superiority of the described sampling techniques over forearm vein blood draws for biomarkers has not been proven and must be done. The use of fluoroscopy exposes the patient to ionized radiation but can be justified the same way as PET research studies. The administration of moderate sedation and monitoring of vital signs by a trained nurse is necessary. The blood draws cannot be performed in a clinic. Risks of vascular injury, although low, are higher than with a forearm vein blood draw. Costs associated with these blood draws will be higher than for forearm phlebotomy. Because ultrasound and fluoroscopy equipment are required, these blood sampling techniques may not be available in underserved regions.

Although there will be initial challenges to the implementation of the described blood sampling techniques, we believe that they may serve to optimize the quality of data derived from blood draws in developing the next generation of blood-based biomarkers in AD research and ultimately the AD clinic.

## Disclosures

PVS did not receive any external funding for this article. PVS has received grants from Valeant Pharmaceuticals and North Carolina Biotech Foundation. PMD did not receive any external funding specifically for this article. PMD has received grants from NIH, ONR, DOD, DARPA, Lilly, USHBC, Cure Alzheimer Fund and ADDF. PMD has received gifts from Karen L Wrenn Trust and Steve Aoki Foundation. PMD has received advisory fees from Otsuka, Prospira, Nutricia, Lumos Labs, UMethod, Transposon, Nestle, and Cornell. PMD is a minor shareholder in Evidation, UMethod, Alzheon, Transposon. PMD serves on the board of AHEL and LLLF. PMD is a co-inventor on several patents in this field.

## Data availability statement

The original contributions presented in the study are included in the article/supplementary material, further inquiries can be directed to the corresponding author.

## Author contributions

PS: Conceptualization, Data curation, Formal analysis, Investigation, Methodology, Project administration, Software, Supervision, Validation, Writing – original draft, Writing – review & editing. PD: Conceptualization, Data curation, Formal analysis, Investigation, Methodology, Project administration, Software, Supervision, Validation, Writing – original draft, Writing – review & editing.
